# Activation of the Nrf-2/HO-1 signalling axis can alleviate metabolic syndrome in cardiovascular disease

**DOI:** 10.1080/07853890.2023.2284890

**Published:** 2023-12-01

**Authors:** Chi Liu, Xingli Xu, Xing He, Junyi Ren, Mingxuan Chi, Gang Deng, Guisen Li, Moussa Ide Nasser

**Affiliations:** a Department of Nephrology, Sichuan Clinical Research Center for Kidney Disease, Sichuan Provincial People’s Hospital, University of Electronic Science and Technology, Chengdu, China; b Chinese Academy of Sciences Sichuan Translational Medicine Research Hospital, Chengdu, China; c Ultrasound in Cardiac Electrophysiology and Biomechanics Key Laboratory of Sichuan Province, Sichuan Provincial People’s Hospital, University of Electronic Science and Technology of China, Chengdu, China; d School of Clinical Medicine, Chengdu Medical College, Chengdu, China; e School of Medicine, University of Electronic Science and Technology of China, Chengdu, China; f Department of Cardiac Surgery, Guangdong Provincial People’s Hospital (Guangdong Academy of Medical Sciences), Southern Medical University, Guangdong Cardiovascular Institute, Guangzhou, Guangdong, China

**Keywords:** Metabolic syndrome, cardiovascular diseases, Nrf2/HO-1, reactive oxygen stress, mitochondrial

## Abstract

**Background**: Cardiovascular disease (CVD) is widely observed in modern society. CVDs are responsible for the majority of fatalities, with heart attacks and strokes accounting for approximately 80% of these cases. Furthermore, a significant proportion of these deaths, precisely one-third, occurs in individuals under 70. Metabolic syndrome encompasses a range of diseases characterized by various physiological dysfunctions. These include increased inflammation in adipose tissue, enhanced cholesterol synthesis in the liver, impaired insulin secretion, insulin resistance, compromised vascular tone and integrity, endothelial dysfunction, and atheroma formation. These factors contribute to the development of metabolic disorders and significantly increase the likelihood of experiencing cardiovascular complications.**Method**: We selected studies that proposed hypotheses regarding metabolic disease syndrome and cardiovascular disease (CVD) and the role of Nrf2/HO-1 and factor regulation in CVD research investigations based on our searches of Medline and PubMed.**Results**: A total of 118 articles were included in the review, 16 of which exclusively addressed hypotheses about the role of Nrf2 on Glucose regulation, while 16 involved Cholesterol regulation. Likewise, 14 references were used to prove the importance of mitochondria on Nrf2. Multiple studies have provided evidence suggesting the involvement of Nrf2/HO-1 in various physiological processes, including metabolism and immune response. A total of 48 research articles and reviews have been used to highlight the role of metabolic syndrome and CVD.**Conclusion**: This review provides an overview of the literature on Nrf2/HO-1 and its role in metabolic disease syndrome and CVD.

## Introduction

Metabolic syndrome (MetS) is a cluster of risk factors, including abdominal obesity, poor glucose tolerance, hypertriglyceridaemia, low high-density lipoprotein (HDL) cholesterol level, and/or hypertension [1,2]. Insulin resistance seems to be a primary mediator of MetS because it is associated with the development of vascular and metabolic dysfunctions, CVD, and type 2 diabetes and occurs in association with the characteristics above. Each MetS component is a risk factor for CVD in and of itself. These risk factors enhance the incidence and severity of CVD when paired with microvascular dysfunction, coronary atherosclerosis, calcification, cardiac dysfunction, myocardial infarction, and heart failure (HF). Subclinical organ damage seems to be the starting point for the processes underlying the increased cardiovascular risk of MetS. As a result, we were interested in the relationship between metabolic disorders and CVD [3–6].

The cap n’ collar basic leucine zipper transcription factors belong to a broad family of transcription factors, including NF-E2 p45-related factors (nuclear factor erythroid 2-related factors Nrf1–3) and transcriptional repressors (Bach1 and Bach2). Nrf2, a member of this family, is a master redox regulator that regulates intrinsic antioxidant genes and phase-II detoxification enzymes [7]. Kelch-like ECH-associated protein 1 (Keap1) is a cysteine-rich inhibitor protein that is covalently linked to the actin cytoskeleton. Under normal conditions, Keap1 acts as an adaptor protein for the Cullin-3 (Cul3)-dependent E3 ubiquitin ligase complex and is responsible for the cytosolic sequestration of Nrf2 [8]. Indeed, Nrf2 has six functional Neh domains that are required for transcriptional activity regulation or degradation. Neh2 was discovered at the N-terminal end and explained Keap1 binding, which requires two specific amino acid sequences in Neh2, ETGE, and DLG. This interaction is required to degrade Nrf2 and Neh6 with a high concentration of serine residues. By interacting with the CREB-binding protein, the Neh4 and Neh5 domains boost Nrf2 transcriptional activity. Neh1 is a domain for DNA binding and dimerization with tiny Maf proteins, followed by the C-terminal Neh3 domain [9–12]. After the N-terminal region, the BTB domain is required to produce Keap1 homodimers and recruit Cul3. Additionally, the intervening region enables Cul3 interactions. The double-glycine mediates Nrf2/Keap1 interaction repeats, a critical characteristic of the Kelch domain [13].

Under normal conditions, Keap1 rapidly promotes Nrf2 ubiquitination and degradation, with a half-life of approximately 20 min [14]. Keap1 inactivation and Nrf2 stabilization occur owing to at least 27 electrophile-modified reactive cysteines implicated in the thiol-rich Keap1 protein and Nrf2 stabilization and increase owing to its 200-min half-life [15,16]. An electrophile or reactive oxygen species (ROS) modifies the –SH groups in Keap1 or phosphorylates Nrf2, enabling Nrf2 to detach from Keap1 and enter the nucleus, where it dimerizes with tiny Maf family members and binds to the conserved antioxidant response element (ARE) sequence. Following its binding to Maf proteins, Nrf2 activates AREs and stimulates genetic transcription [17]. Similarly, numerous cytoprotective genes are regulated by the Nrf2/Keap1 pathway, including antioxidant genes, detoxifying molecules, genes encoding enzymes involved in glutathione production, and proteins that influence the expression of other transcription factors or growth factors [18].

As one of the downstream effectors of Nrf2, haem oxygenase (HO) comprises three isozymes (HO-1, HO-2, and HO-3). HO-1 is a 32-kDa inducible protein that can be significantly enhanced by haem, nitric oxide (NO), heavy metals, growth factors, cytokines, and altered lipids. By decomposing haem with NADPH cytochrome P450, HO-1 generates iron ions, biliverdin, and carbon monoxide (CO) [19]. Furthermore, biliverdin is rapidly converted to bilirubin by biliverdin reductase and acts as an effective antioxidant. Other HO-1 products protect tissue by regulating critical biological processes, such as inflammation, apoptosis, proliferation, fibrosis, and angiogenesis [20].

Taken together, activation of the Nrf2/HO-1 axis reduces CVD risk factors by restoring metabolic balance, whereas inhibition of Nrf2 results in metabolic imbalance, thus precipitating CVD.

## 1. Glucose regulation

### 1.1. ROS production is a key factor in CVD

Although several additional inducers exist, oxidative stress is an essential pathophysiological mechanism in heart and vascular diseases. Numerous studies have demonstrated that oxidative stress plays a role in cardiac dysfunction and myocardial apoptosis, contributing to the etiology of HF associated with left ventricular dysfunction. Similarly, the overproduction of ROS or reactive nitrogen species(RNS) has been implicated in the pathophysiology of ischaemic myocardial damage and consequent cardiac dysfunction, with detrimental effects on myocardial calcium management, apoptosis, cardiac remodelling, ventricular hypertrophy, arrhythmia, and necrosis [21–24].

Furthermore, the proper functioning of pro-oxidant and antioxidant systems is of utmost importance in maintaining oxidative balance, as they play a crucial role in regulating the generation and elimination of ROS in specific areas. ROS regulates various cellular processes, including endothelial and smooth muscle cells’ growth, proliferation, and migration. Additionally, ROS is involved in critical physiological functions such as angiogenesis, apoptosis, vascular tone regulation, host defence mechanisms, and maintenance of genomic stability. Activating the redox pathway can lead to protective effects, such as antioxidative capacity, even at low concentrations. However, it is postulated that generating ROS exceeds the antioxidant capacity. In this scenario, there is a manifestation of direct and enduring oxidative harm to macromolecules, alongside endothelial dysfunction and disturbance of the redox-dependent vascular wall axis, resulting in the development of atherosclerosis. The Nrf2/HO-1 pathway provides direct cellular protection against oxidative stress induced by high glucose levels through activating antioxidant enzymes and modulation of glucose metabolism, manifesting cytoprotective effects [22,25].

### 1.2. β-cell proliferation and insulin signalling

Nrf2 activation has been established in animals and is critical for insulin signalling [26,27]. Moreover, to mitigate the abnormalities induced by oxidative stress, the enzymes HO-1 and Mn-SOD, targeted by the transcription factor Nrf2, enhance insulin sensitivity by suppressing the activation of PKC and JNK resulting from mitochondrial dysfunction. Following this, there is an increase in serine phosphorylation IRS-1, while tyrosine phosphorylation of IRS-1 is suppressed. The sequence mentioned above acts as an insulin signaling regulator, thereby impacting glucose metabolism. Moreover, it has been observed that Nrf2 has the potential to directly augment insulin signalling, although the precise mechanism by which this occurs remains unidentified [28]. However, it has been claimed that insulin and its effector Akt/PKB limit Nrf2 nuclear translocation by phosphorylating Nrf2 [29]. Likewise, Nrf2 activation is essential for normal and ChREBP-augmented glucose-stimulated β-cell proliferation, and Nrf2 overexpression is sufficient to increase human β-cell proliferation:

- ChREBPα induces Nrf2 by inhibiting Keap1 activity.
- Nrf2 binds to AREs and increases the expression of antioxidant and metabolic enzyme genes.
- Elevated mitochondrial content and activity drive abundant ATP production and anabolic metabolism, increasing glucose-stimulated β-cell proliferation. The feedback loop contributing to the maintenance of this anabolic state might be inhibiting the Keap1–Nrf2 interaction via the disruption of cysteine residues by electrophiles [30,31].

### 1.3. Glycolysis

Nrf2 regulates the expression of the miR-106b-25 cluster through the stimulatory action of oxidized phospholipids (oxPAPC). MiR-93, the predominant microRNA within this particular cluster, plays a significant role in regulating endothelial glycolysis and quiescence. The expression levels of microRNAs (miR-106b, miR-93, and miR-25) exhibit an upregulation in patients diagnosed with coronary artery disease who have undergone treatment with oxidized 1-palmitoyl-2-arachidonoyl-sn-glycero-3-phosphorylcholine (oxPAPC) [32]. The cytotoxicity of glucose deprivation arises due to the essential nature of glycolysis and continuous glucose supply for the proper functioning of endothelial cells [33]. Similarly, the quiescent endothelium may be encouraged to form a new vascular network responding to local nutrition and oxygen deprivation or neovascularization triggered by Nrf2-mediated oxPAPC [34,35].

Also, glycolysis is essential for the proliferation of endothelial cells and the formation of new blood vessels (angiogenesis). Endothelial cells produce more than 85% of adenosine triphosphate (ATP) through glycolysis in cancer cells. This rate of ATP production is observed to double as the cells undergo a transition from a state of quiescence to active proliferation [36,37].

## 2. Cholesterol regulation

Nrf2 activation may act as an anti-obesity factor by adversely regulating lipid production. Numerous studies have demonstrated the significance of Nrf2 in adipocyte lipid metabolism [28,38]. Likewise, the activations of Nrf2 by carnosic acid and carnosol resulted in a significant increase in the intracellular level of total glutathione (GSH). Consequently, the inhibition of preadipocyte differentiation and adipogenesis leads to a reduction in obesity [39]. Similarly, oltipraz and the oleanolic triterpenoid 1-[2-cyano-3,12-dioxooleana-1,9(11)-dien-28-oyl] imidazole, which are two well-characterized Nrf2 activators, have been demonstrated to protect against HFD-induced increases in body weight, adipose mass, and hepatic fat accumulation [40,41]. Similarly, research in the livers of mice revealed that the Nrf2 system is involved in suppressing lipid synthesis and metabolic enzymes, such as ATP-citrate lyase [42].

Elevated cholesterol levels have been found to induce oxidative stress and disrupt the energy metabolism of cardiomyocytes, ultimately contributing to the development of CVD. The association between HO-1/emopamil-binding protein (EBP) safeguards the myocardium by mitigating the detrimental effects of oxidative stress-induced and systolic cardiac dysfunction. EBP exhibits an interaction with HO-1. The interaction between HO-1 and endoplasmic EBP plays a crucial role in lipid metabolism, the breakdown of surplus cholesterol, and mitigating the impact of cholesterol on oxidative stress. The activation of the Akt and Nrf2/mTOR pathways leads to an upregulation of the expression of EBP and HO-1 in response to cholesterol stimulation [43]. In a similar vein, macrophages are known to internalize modified lectin-like oxidised low-density lipoprotein (LDL) receptor 1, toll-like receptor (TLR) 4, and chemokine (C-X-C motif) ligand 16. Lysosomal acid lipase hydrolyzes excess-free cholesteryl esters (CEs) within internalised modified LDL particles, producing free cholesterol (FC). FC is exported from the cellular interior by utilizing ATP-binding cassette (ABC) transporters, specifically ABCA1 and ABCG1. ABCA1 and ABCG1 facilitate the transportation of lipids to apoA-1 and HDL, respectively. The enzyme ACAT1 is responsible for re-esterifying FC into CE within the endoplasmic reticulum (ER). These cholesteryl esters are subsequently stored as lipid droplets in the cytoplasm. Foam cells are generated through the accumulation of lipid droplets, which subsequently trigger the activation of Nrf2 and its associated antioxidant proteins, such as HO-1, peroxiredoxins, and GPx1. The activation of Nrf2 has the potential to either increase or decrease the expression of lipoprotein uptake receptors in the presence of atherogenic conditions. Additionally, it can potentially enhance the synthesis of ABC transporter proteins through scavenger receptors (SRs) like SR-A and SR-B (CD36) and lectin-type oxidation, thereby facilitating cholesterol efflux [44,45].

Additionally, CO and bilirubin generated by HO-1 sustain preadipocytes in an early differentiation stage, i.e., smaller adipocytes are considered healthy and insulin-producing. Further, biliverdin and bilirubin reduce obesity by modifying hypertrophic adipocytes and inhibiting ROS-mediated adipogenesis. Moreover, HO-1 induces the formation of epoxyeicosatrienoic acid and PGC-1, which enhance mitochondrial biogenesis and integrity while limiting the accumulation of fatty acids (FAs) and obesity. Indeed, PGC-1, a crucial regulator of energy metabolism, targets SIRT3, a mitochondrial deacetylase, and promotes mitochondrial biogenesis, ROS suppression, and FA oxidation [46].

**Figure 1.**
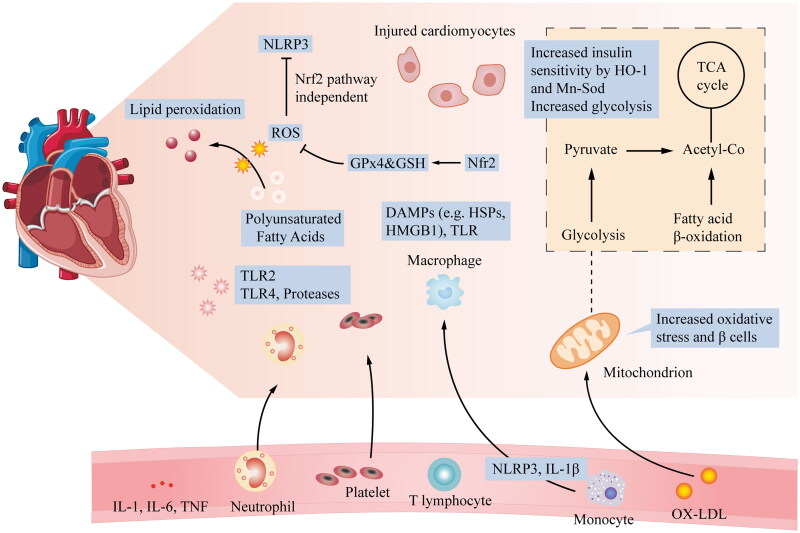
Relationship of glycolysis, β-cell proliferation, insulin signalling, and ROS production with CVD: after myocardial injury, many local processes are activated. ROS and cytokines are released, and neutrophils and monocytes accumulate in the blood vessels, leading to acute myocardial injury. At the cardiomyocyte level, important mechanisms include increased glycolysis, energy changes, increased oxidative stress, and β-cell proliferation. Additionally, to alleviate oxidative stress-induced abnormalities, HO-1 and Mn-SOD, which are Nrf2-targeted enzymes, increase insulin sensitivity by inhibiting PKC and JNK activation caused by a mitochondrial malfunction.

Lipid peroxidation is a reaction in which ROS and RNS combine with polyunsaturated FAs in the plasma and organellar membranes to form lipid peroxides, a critical starter of the ferroptosis cascade [47,48]. Nrf2 acts as a master regulator of the antioxidant response, regulating the activity of many ferroptosis- and lipid peroxidation-related proteins. These targets are broadly classified as iron/metal, intermediate, and GSH synthesis/metabolism targets. GPx4 uses GSH to convert lipid peroxides to their alcohol form, which is a critical step in avoiding ferroptosis (Figure 1) [49].

## 3. Mitochondrial and Nrf2

The binding of lipopolysaccharide (LPS) to Toll-like receptors (TLRs) initiates the generation of ROS within the mitochondria by causing damage to mitochondrial DNA (mtDNA), resulting in either inflammation of the heart or fragmentation of the mitochondria. In addition, oxidative stress can cause aberrant mitochondrial morphology, which may result in an increase in ROS production. Reduced mitochondrial membrane potential results in decreased cellular ATP levels and increased ROS production [50–52]. The activity of Nrf2 in the mitochondria, which is responsible for ROS generation, coincides with the unique way in which cells and tissues function. Mst1 and Mst2 are recruited to the mitochondria in macrophages in a ROS-dependent manner and activate Nrf2 through Keap1 phosphorylation [53]. Additionally, a study demonstrating that MEF2D controls Nrf2 mRNA levels in retinal epithelial cells supports the MEF2 regulatory mechanism for Nrf2 mRNA. The method by which complex I activity maintains extracellular signal-regulated protein kinase (ERK) 5 expression remains unknown [54].

Mitochondrial quality control (mQC) is critical for the proper functioning of cells and the mitochondria. In the absence of mQC, fragmented mitochondria aggregate in the cells and form ROS. NQO1, HO-1, and p62 act as Nrf2 target genes and contribute to mQc [55]. Under oxidative stress conditions, Nrf2 regulates the expression of PINK1, which regulates complex I activity by phosphorylating the complex I subunit NdufA10 and activating tricornered/NDR kinase [56–58].

The restoration of mitophagy-related gene expression, including p62, Atg9b, and Ulk1, occurs when Nrf2 is overexpressed or pharmacologically activated. However, there is a decrease in mitochondrial fragmentation and the interferon response observed in mouse embryonic fibroblasts (MEFs) lacking N-glycanase 1 (NGLY1). This implies that Nrf2 may be able to induce the activation of mQC in cellular contexts where NGLY1 is absent and potentially in individuals who have NGLY1 deficiency [59]. HO-1 is a critical downstream target of Nrf2. The following data demonstrate that HO-1 protects the heart by regulating mitochondrial biogenesis, dynamics, and mitophagy:

- Overexpression of HO-1 protects against myofibril reorganization, mitochondrial fragmentation, mtDNA depletion, and mitochondrial damage in autophagic vacuoles.
- The expression of HO-1 increases the expression of NRF-1, PGC1, and TFAM, which influence mitochondrial biogenesis.
- The expression of HO-1 inhibits the increased expression of Fis1 and induces the expression of the fusion mediators Mfn1 and Mfn2.
- The expression of HO-1 also increases the expression of two key mediators of the mitophagy pathway: PINK1 and parkin.

Redox homeostasis is critical for cellular function and the pathophysiology of CVD, with excessive ROS resulting in oxidative stress and modest ROS levels benefiting cells by activating Nrf2. The heart is primarily a postmitotic organ that generates energy through FAs. As a result, the heart has more mitochondria than other organs, and maintaining redox balance and mitochondrial integrity is critical for heart physiology [60]. In addition, Nrf2 is localized to the mitochondria in the rat heart and has been shown to protect the mitochondria from oxidative damage caused by H_2_O_2_ [61]. The G-quadruplex structure of mRNA and elongation factor 1-alpha, which have been shown in HEK293 cells, promote H_2_O_2_-induced Nrf2 translational activation [62].

On the other hand, the myocardial activating transcription factor known as Nox4 exhibits an increase in expression in response to acute exercise-induced stress. This upregulation subsequently triggers the activation of the Nrf2/ARE signalling pathway. In addition, it has been observed that PRXIII, TRXR2, and SOD2 can function as target genes of Nrf2. These genes are crucial in diminishing mitochondrial ROS, sustaining cardiac function, and enhancing exercise performance [63,64].

**Figure 2.**
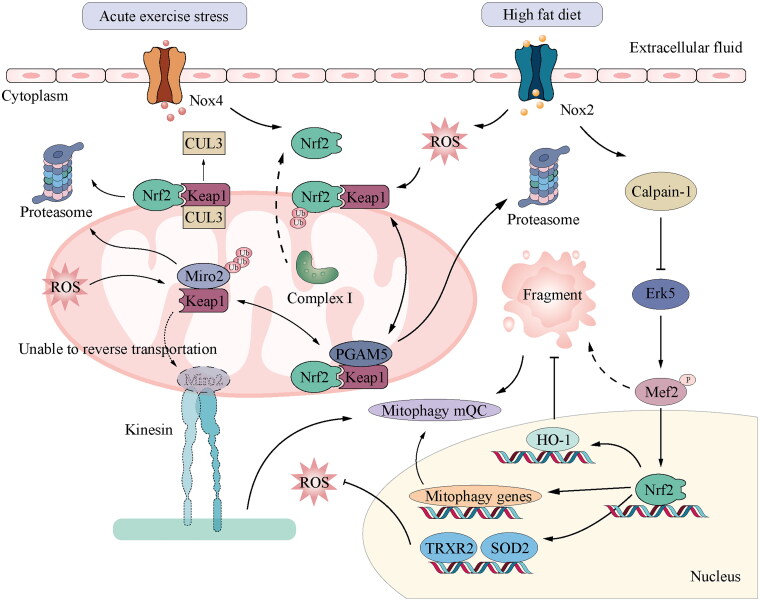
Function of mitochondrial regulation through Nrf2 signalling in CVD: ubiquitination regulation of Nrf2 is key to the cellular response to oxidative and electrophilic stresses. Nrf2 is polyubiquitinated by the Keap1–Cul3 complex in the basal state without oxidative or electrophilic stress. Cul3 is a ubiquitin ligase, while Keap1 is a substrate adapter. This polymerisation causes Nrf2 to be degraded by the proteasome. In the mitochondria, PGAM5 and Nrf2 bind to the monomer of the Keap1 dimer in a mutually exclusive manner. Nrf2 and PGAM5 regulate the activity of the Keap1–Cul3 E3 ubiquitin ligase, thus protecting Miro2 from abnormal degradation. In contrast, the activation of Nox2 induced by a high-fat diet induces ROS production, leading to calpain-1 mediated destruction of ERK5 and mitochondrial dysfunction by lowering the ERK5-MEf2-PGC α pathway. Similarly, acute exercise stress can upregulate the level of Nox4 in the myocardium and activate Nrf2/ARE signal transduction of Nrf2 target genes, such as TRXR2 and SOD2, which can reduce mitochondrial ROS and maintain cardiac performance. Nrf2, nuclear factor erythroid 2-related factor 2; Keap1, Kelch-like ECH-associated protein 1; Cul3, Cullin-3.

Furthermore, the presence of metabolic stressors such as obesity, hyperglycaemia, hyperlipidaemia, chronic inflammation, and mitochondrial dysfunction leads to the accumulation of ROS, ultimately contributing to the development of heart failure in the ageing population. While the activation of ATF4 and the maintenance of cellular redox homeostasis can be attributed to ER or mitochondrial stress, it is essential to note that chronic stress leads to the activation of the CHOP-induced apoptotic pathway and the disturbance of mitochondrial homeostasis [65]. Nrf2 activates cytoprotective genes, including the cystine transporter xCT, and cooperates with ATF4 to maintain the redox balance and inhibit ATF4-mediated CHOP expression. The formation of the Nrf2/KEAP1/PGAM5 complex occurs within the mitochondria, and the retrograde transport of mitochondria is impeded when either Nrf2 or PGAM5 is absent due to the inhibition of proteasome activity. The dyskinesia in question is primarily attributed to the aberrant degradation of Miro2, a mitochondrial GTPase that serves as a link between mitochondria and microtubules. Miro2 is a GTPase protein located in the mitochondria, which serves as a connecting link between mitochondria and microtubules [66] (Figure 2).

## 4. CVD

### 4.1. Atherosclerosis

Atherosclerosis is characterized by the deposition of cholesterol esters, inflammatory cells, smooth muscle cells (SMCs), and extracellular matrix components like collagen and elastin within the arterial wall. The progression of atherosclerosis leads to the constriction of blood flow, haemorrhaging caused by rupture, and myocardial infarction resulting from thrombosis. Several risk factors play a role in the development of atherosclerosis. These include an increased level of LDL cholesterol in the bloodstream, a decreased level of HDL cholesterol in the bloodstream, diabetes, hypertension, smoking, advancing age, and oxidative stress. The promoter’s (GT)n segment, characterized by a high degree of polymorphism, plays a crucial role in regulating the basal and stimulated expressions of HO-1 *in vivo*. This regulatory mechanism can significantly impact the anti-inflammatory and pro-angiogenic functions associated with HO-1 [67,68].

Pro-oxidant and pro-atherogenic stimuli, such as ox-LDL and oxPAPC, can potentially induce the expression of Nrf2-regulated HO-1 and various other antioxidant genes in vascular cells. This, in turn, can lead to a decrease in ROS production. In contrast, the expression of HO-1 is significantly upregulated in macrophages present in atherosclerotic lesions. Multiple studies have demonstrated that it hinders the progression of atherosclerosis in these cells through the modification of lipid loading and, to some extent, by enhancing the expression of SR-A. The upregulation of HO-1 expression results in a decrease in foam cell formation and alters the levels of low-d.

LDL and HDL receptors, specifically scavenger receptor class A (SR-A). Similarly, the antioxidant activity is notably augmented by the enzymatic byproducts of HO-1, namely biliverdin and ferrous iron (Fe^2+^). Consequently, ROS production and inflammatory processes, such as the downregulation of cell adhesion molecules and the diminished release of inflammatory proteins, namely MCP-1 and IL-8, are reduced. Bilirubin is produced through the oxidative conversion of biliverdin and can potentially impede the oxidation of LDL and other lipids while also functioning as an oxygen radical scavenger [69–71].

Carbon monoxide is an enzymatic byproduct of HO-1, which plays a critical role in atherosclerosis. It may stimulate the p38 mitogen-activated protein kinase (MAPK) signalling pathway [72,73]. In addition, CO has anti-inflammatory properties. It has been shown to influence the response of monocytes/macrophages to bacterial LPS, block macrophage TLR signalling through downstream activation of NF-kB, and suppress GM-CSF production by altering macrophage differentiation [74,75]. Interestingly, the MAPK signalling pathway also mediates anti-inflammatory actions involving ROS formation, as ROS causes an increase in CO in macrophages [76].

It may also bind to and activate guanylate cyclase, increasing intracellular cyclic guanosine monophosphate (cGMP) levels [77,78]. CO has also been shown to be helpful in balloon injury models. These effects involve the stimulation of the p38 MAPK pathway, which is mediated by cGMP and requires guanylate cyclase activity [79]. The function of Nrf2 in promoting atherosclerosis can be described as follows:

- Nrf2 expression likely enhances liver lipogenesis, increasing plasma non-HDL cholesterol levels in ApoE-null mice [80,81].
- Nrf2 promotes foam cell formation via upregulation of the CD36 SRs and increases IL-1 expression in macrophages, resulting in increased monocyte migration to lesions [82,83].
- Nrf2 may govern macrophage development into subtypes Mox and Mhem, distinct from the standard M1 and M2 [84–86].

### 4.2. Hypertension

Oxidative stress has been linked to vascular damage caused by hypertension, including endothelial dysfunction, vascular remodelling, and enhanced contractility [87,88]. Numerous enzymes in the circulatory system, including NADPH oxidase, uncoupled endothelial NO synthase, mitochondrial electron transport chain oxidase, and xanthine oxidase, may generate ROS [89].

These mechanisms are dysregulated in patients with hypertension, resulting in increased ROS production, oxidative stress, and decreased antioxidant capacity. Nrf2 is an antioxidant regulator in patients with hypertension, lowering ROS bioavailability and boosting NO synthase-induced NO production [90]. Nonetheless, Nrf2 activators can inhibit Ang II vasoactive effects, improve endothelial function, and normalise vascular contraction. These results indicate that the downregulation of vascular Nrf2 may result in oxidative stress associated with vascular dysfunction in patients with hypertension and that Nrf2 activators mitigate these deleterious effects. These vasoprotective effects may be clinically beneficial for treating hypertension-related vascular damage by reducing oxidative stress and increasing vascular function [91,92].

Moreover, several medicines that activate Nrf2 have been demonstrated to exert potential vasoprotective effects in patients with hypertension. Nrf2 is dysregulated in hypertension models, and Nrf2 activators have been shown to prevent blood pressure increases. In rats with renal dopamine 2 receptor impairment, bardoxolone normalized high blood pressure and decreased Nrf2 expression in the kidneys [93]. Hepatic Nrf2 expression and antioxidant gene levels are increased in fermented barley-fed SHRSP, whereas the rise in blood pressure is attenuated [94].

### 4.3. Myocardial ischaemia-reperfusion (IR)

The myocardium is susceptible to ischaemic injury because oxygen absorption is approximately 80% at any moment during cellular perfusion. Cardiomyocytes cannot considerably improve oxygen intake from arterial blood in instances of severe vascular constriction, such as atherosclerotic plaque, thrombosis, or vasospasm. An inflammatory cascade is triggered when blood flow is restored, oxidative stress increases, and antioxidant defences are overwhelmed. This results in cardiac dysfunction due to cell death or damage, resulting in the oxidation of DNA, promotion of chain reactions of membrane lipid peroxidation, and changes in membrane fluidity [95–97]. Additionally, neutrophil migration from the vasculature to the tissue leads to an aggravation of tissue damage caused by ischaemia, even though oxygen and trophic chemicals are supplied to cells during reperfusion [98,99]. In IR-damaged vascular tissue, superoxide anions, a type of significant ROS, are generated by the catalysis of NADPH oxidase in neutrophils or by leakage of the electron transport chain in the mitochondria. IR-induced oxidative stress results in myocardial apoptosis, which may be mitigated by free radical scavengers [100]. Similarly, it has been found that its enhanced translation rapidly activates Nrf2 and that acute Nrf2 activation has a cardioprotective effect in the rat heart during IR.

Furthermore, increasing Tsg101 levels may protect against myocardial IR damage by activating the p62–Keap1–Nrf2 signalling pathway. Additionally, in animals subjected to IR damage or pressure overload, therapies against ER stress and Nrf2 activation have been shown to reduce the number of myocardial infarcts and cardiac hypertrophy during the transition to HF [101]. In contrast, blocking the left anterior descending coronary artery for 30 min lowers Nrf2 nuclear protein levels in the rat heart, and this effect is blocked by myocardial ischaemia preconditioning (IPC). This implies that the duration of the preceding ischaemia phase is crucial for Nrf2 to launch antioxidant defences against reperfusion-induced oxidative stress and that IPC may serve as a warning signal to activate Nrf2 before a lengthy ischaemic event [102].

Similarly, arctigenin (ATG) protects cardiac tissue against IR damage by increasing Trx1, SOD, and GSH-Px activities while decreasing MDA levels. Following myocardial IR, ATG increases Nrf2 and Nox1 protein expression in the ischaemic heart. Nrf2 induces Trx1, SOD, and GSH-Px expression. ATG also reduces the severity of ventricular arrhythmias at moderate or high doses, as evaluated by the frequency and duration of ventricular fibrillation and tachycardia [103].

### 4.4. Diabetic cardiomyopathy (DCM)

Diabetes is a chronic metabolic disorder that is characterized by abnormal glucose metabolism. It may result in cardiovascular problems and heart remodelling, beginning with myocardial hypertrophy and apoptosis and advancing to left ventricular diastolic insufficiency, which can result in HF in extreme instances. Metabolic dysregulation, aberrant calcium homeostasis, cardiac autonomic neuropathy, insulin resistance, myocardial hypertrophy, and fibrosis are hallmark features of DCM, with myocardial hypertrophy and fibrosis being especially significant [104]. Current evidence shows that hyperglycaemia-induced oxidative stress and subsequent inflammatory and nitrifying stress play critical roles in the onset and progression of DCM [105]. Similarly, during the early stages of diabetes, reactive expression of myocardial Nrf2 is increased, along with the mRNA levels of downstream genes NQO1, HO-1, and GST, to counteract the early oxidative damage in patients with diabetes and protect myocardial cells from death caused by high glucose levels. HO-1 has been shown to play a critical role in the protective mechanism of Nrf2 against oxidative damage and cardiac hypertrophy. However, in the late stages of diabetes, the antioxidant capacity of the heart is further compromised, and Nrf2 and Brg1 are inactivated owing to increased ROS/RNS formation. Nrf2 expression is considerably lower in the heart, decreasing HO-1 synthesis. During this stage, myocardial hypertrophy and apoptosis are increased, the glucose metabolism of the heart is substantially impaired, and the progression of DCM is hastened. Additionally, oxidative stress may result in insulin resistance and impede insulin-induced glucose absorption by adult cardiomyocytes. Nrf2 activation inhibits the activity of ERK generated by oxidative stress, reverses oxidative stress-induced insulin resistance, and stimulates glucose uptake by adult cardiomyocytes [106–108]. These findings imply that reversing the decreased expression and activity of Nrf2 in the late stage may effectively inhibit the occurrence and development of DCM. A new treatment method for myocardial insulin resistance and DCM can be developed with the inhibition of Nrf2.

### 4.5. Cardiac senescence

The process of cardiac ageing detrimentally affects both the systolic and diastolic functioning of the heart, thereby augmenting its susceptibility to injury. The mechanism of cardiac ageing remains a topic of debate within the academic community. However, several prominent perspectives highlight three key factors that are believed to play a significant role: oxidative stress damage, inflammatory response, and decreased autophagy. In an experimental model of ischaemia-reperfusion-induced injury in rat hearts, administering Ginkgo biloba leaf extract (EGb 761) increased serine-threonine protein kinase (Akt) phosphorylation. This activation of Akt subsequently facilitated the translocation of Nrf2 into the nucleus, leading to an upregulation of HO-1 expression. Indeed, HO-1 can reduce the degradation of haemoglobin, producing bilirubin, CO, and Fe^2+^. This enzymatic process contributes to the reduction of oxidative stress and inflammatory reactions and the inhibition of apoptosis in myocardial cells [109]. A high-fat diet al.so induced heart damage in wild-type (Nrf2^+/+^) and Nrf2^-/-^ mice, and ethanol boosted Nrf2 protein expression and antioxidant activity downstream. The GCL catalytic component GCLC and the regulatory subunit GCLM were upregulated, reducing ROS production. Moreover, Nrf2 reduced the levels of pro-inflammatory cytokines, such as TNF-α, IL-1B, IL-18, and IL-6, preventing heart damage induced by high-fat diets [110].

Additionally, Nrf2 deficiency enhanced the accumulation of harmful ubiquitinated proteins generated by doxorubicin (DOX) in the heart, resulting in increased expression of DOX-induced soluble and insoluble LC3-II P62 proteins, as well as increased DOX-induced cardiac autophagy damage. By increasing the autophagy clearance of ubiquitinated protein aggregates, Nrf2 activation ameliorates DOX-induced cardiac autophagy injury [111]. These findings imply that Nrf2 may help postpone cardiac ageing by modulating oxidative stress, inflammatory response, and autophagy indirectly or directly.

### 4.6. Vascular calcification

Vascular calcification mainly manifests as decreased vascular wall compliance and increased stiffness, which is one of the essential reasons for the high incidence and mortality of CVD and cerebrovascular diseases and the common pathophysiological basis of hypertension, atherosclerosis, vascular injury, and ageing [112,113].

**Figure 3.**
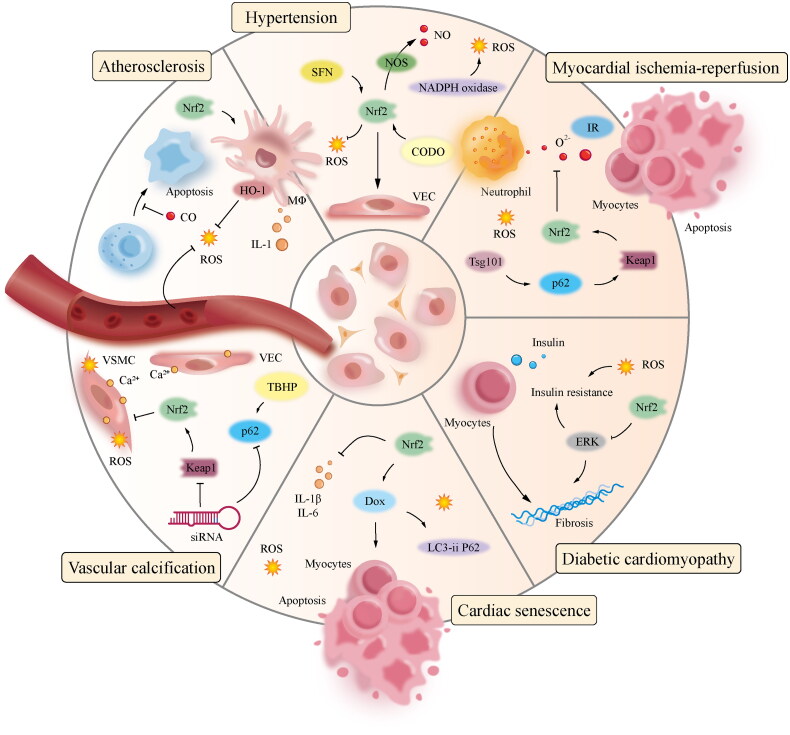
Regulation of Nrf2 in CVD.

The inorganic phosphate levels can potentially induce oxidative stress and promote calcification in vascular smooth muscle cells (SMCs). This leads to an upregulation of the autophagy-related protein P62 and a decrease in Keap1 levels. In order to mitigate the accumulation of reactive oxygen species (ROS) and calcium deposits within muscle cells, the compound tert-butyl hydro benzoyl facilitates the translocation of the nuclear factor erythroid 2-related factor 2 (Nrf2) into the cell nucleus, thereby enhancing the expression of P62 and Keap1. Similarly, suppressing Nrf2 and P62 using siRNA leads to increased reactive oxygen species (ROS) and calcium buildup in muscle cells. Activating the Keap1/Nrf2/P62 signalling pathway could potentially mitigate the calcification of muscle cells induced by high phosphate levels [114]. Similarly, rosiganoic acid plays an antagonistic role in vascular calcification by regulating the Nrf2 signal transduction pathway. When Nrf2 was silenced in a previous study, the protein expressions of Nrf2, NQO-1, HO-1, and osteoprotegerin in the interference and interference plus rosiganoic acid groups were significantly lower than those in the model and normal groups. Calcification was significantly higher in the interference and interference plus rosmarinic acid groups than in the model and administration groups, suggesting that the Nrf2 pathway plays a decisive role in vascular calcification [115]. These findings suggest that Nrf2 plays an essential role in inhibiting vascular calcification. The mechanism by which Nrf2 regulates CVD is shown in Figure 3.

## 5. Perspectives

Multiple components of metabolic syndrome contribute to the buildup of epicardial adipose tissue. The presence of epicardial adipose tissue, along with the associated pathological inflammation and endothelial dysfunction, can lead to the development or exacerbation of CVDs in individuals diagnosed with metabolic syndrome. Hence, it can be inferred that targeting epicardial adipose tissue holds potential as a novel approach for both preventing and treating cardiovascular diseases. The management of metabolic syndrome and CVD through pharmacological interventions aimed at enhancing metabolism and preventing cardiovascular conditions is closely associated with the intervention of epicardial adipose tissue [116]. The administration of statins has decreased coronary artery calcification by reducing the epicardial adipose tissue volume and alleviating the inflammatory state. Notably, there is no statistically significant correlation between the extent of reduction in LDL cholesterol levels and the effect above. Glucagon-like peptide-1 receptor agonists have been shown to mitigate the risk of CVD through their capacity to decrease the thickness of epicardial adipose tissue [117,118]. Likewise, sodium-glucose cotransporter 2 inhibitors have been found to possess the ability to reduce the thickness of epicardial adipose tissue, enhance glucose uptake within this tissue, diminish the secretion of pro-inflammatory chemokines, promote the cellularization of epicardial adipose tissue, and subsequently mitigate the likelihood of cardiovascular diseases. The potential applications of epicardial adipose tissue in preventing and managing metabolic syndrome and CVD are extensive. Enhancing the pathological condition of epicardial adipose tissue can potentially alleviate insulin resistance and chronic inflammation in individuals, thereby facilitating the amelioration of metabolic syndrome and mitigating and managing complications associated with metabolic syndrome.

Adipose tissue releases a significant quantity of non-esterified fatty acids, hormones, and pro-inflammatory cytokines, which can impede the transport and phosphorylation of glucose. This inhibition contributes to the development of insulin resistance and plays a role in endothelial dysfunction and cardiovascular diseases. Hence, obesity constitutes a risk factor for the development of atherosclerosis. The correlation between mental stress and heightened morbidity and mortality rates of cardiovascular diseases has been well-established. One of the potential mechanisms underlying this relationship is the detrimental impact of mental stress on endothelial cells. During periods of stress, the activation of sympathetic nerves results in the elevation of local norepinephrine levels. This increase in norepinephrine leads to the up-regulation of adhesion molecules and chemokines by binding to alpha-adrenergic receptors located on endothelial cells. Additionally, norepinephrine binds to receptors on macrophages and smooth muscle cells, thereby intensifying inflammatory effects. Collectively, these processes contribute to the augmentation of endothelial permeability and dysfunction, ultimately culminating in chronic vascular inflammation. Additional acknowledged factors contributing to cardiovascular health comprise a sedentary way of life while engaging in routine physical exercise has been shown to mitigate the likelihood of developing cardiovascular disease. However, it is possible that focusing on the Nrf2/HO-1 axis as a means to address endothelial dysfunction could play a role in promoting it.

## Conclusions

The Nrf2/HO-1 signalling axis has a complex regulatory mechanism in CVD and plays anti-inflammatory and antioxidant roles. It reduces mitochondrial damage and regulates intracellular Ca^2+^ flow and cell death, which are indispensable signalling axes for protecting cells from oxidative stress. Therefore, activation of the Nrf2/HO-1 signalling axis can mitigate the effects of metabolic dysfunction in CVD. Notably, whether Nrf2 activation is good is not well-defined. Nrf2 plays a complex and diverse role in human diseases (i.e. preventing chronic diseases and cancer, helping cancer cells survive, or making tumour cells resistant to radiation and chemotherapy). The dual roles of Nrf2 and HO-1 indicate that the regulatory mechanism of this signalling axis has not yet been fully explored. In the future, researchers should further explore the pathogenesis of Nrf2/HO-1 in CVD, provide a molecular basis for targeted drug therapy research, and realize this axis’s potential value in clinical drug research.

## Data Availability

Not applicable.
